# A Citizen-Science Study Documents Environmental Exposures and Asthma Prevalence in Two Communities

**DOI:** 10.1155/2016/1962901

**Published:** 2016-11-24

**Authors:** Samantha Eiffert, Yomi Noibi, Stephen Vesper, Jonathan Downs, Florence Fulk, Juanita Wallace, Melanie Pearson, Andrea Winquist

**Affiliations:** ^1^Department of Environmental Health, Rollins School of Public Health, Emory University, 1518 Clifton Road NE, Atlanta, GA 30322, USA; ^2^ECO-Action, 250 Georgia Avenue, SE 309, Atlanta, GA 30312, USA; ^3^National Exposure Research Laboratory, United States Environmental Protection Agency, 26 West M. L. King Drive, Cincinnati, OH 45268, USA

## Abstract

A citizen-science study was conducted in two low-income, flood-prone communities in Atlanta, Georgia, in order to document environmental exposures and the prevalence of occupant asthma. Teams consisting of a public-health graduate student and a resident from one of the two communities administered a questionnaire, inspected residences for mold growth, and collected a dust sample for quantifying mold contamination. The dust samples were analyzed for the 36 molds that make up the Environmental Relative Moldiness Index (ERMI). Most residents (76%) were renters. The median duration of residence was 2.5 years. Although only 12% of occupants reported a history of flooding, 46% reported at least one water leak. Homes with visible mold (35%) had significantly (*P* < 0.05) higher mean ERMI values compared to homes without (14.0 versus 9.6). The prevalence of self-reported, current asthma among participants was 14%. In logistic regression models controlling for indoor smoking, among participants residing at their current residence for two years or less, a positive association was observed between asthma and the homes' ERMI values (adjusted odds ratio per unit increase in ERMI = 1.12, 95% confidence intervals (CI): 1.01–1.25; two-tailed *P* = 0.04). Documentation of the exposures and asthma prevalence has been presented to the communities and public officials. Community-based organizations have taken responsibility for planning and implementing activities in response to the study findings.

## 1. Introduction

The prevalence of asthma has nearly doubled in the US since 1980 resulting in about 26 million current persons with asthma [1, 2]. Therefore, asthma is a disease of growing concern in communities, especially socially and economically disadvantaged communities, which often experience a higher prevalence of asthma than the communities surrounding them [3, 4]. The English Avenue and Vine City areas in Atlanta, Georgia, are two such communities.

Because of their concern, members of these two communities became actively engaged in expressing the need for accurate information about their environment, especially resulting from the flooding of Proctor Creek that runs through this part of Atlanta. Previous flooding events had resulted in the water from the creek entering into the some of the homes in the community. Citizens contacted community-based organizations, universities, the city of Atlanta, and government agencies to voice their need for accurate data about the prevalence of asthma and the housing conditions in their communities. As a result, a study was conducted to document the prevalence of asthma and the environmental exposures that might be associated with asthma, including cigarette smoke, sources of allergens, and mold [5]. The resulting data and public meetings relating to study finding should allow governmental and nongovernmental organizations and the residents themselves to begin the process of reducing these exposures.

## 2. Materials and Methods

The protocol for this study was evaluated by the Emory University Institutional Review Board (IRB) and was determined to be a quality improvement study, not requiring IRB approval. Informed consent was obtained from all study participants.

**Figure 1:**
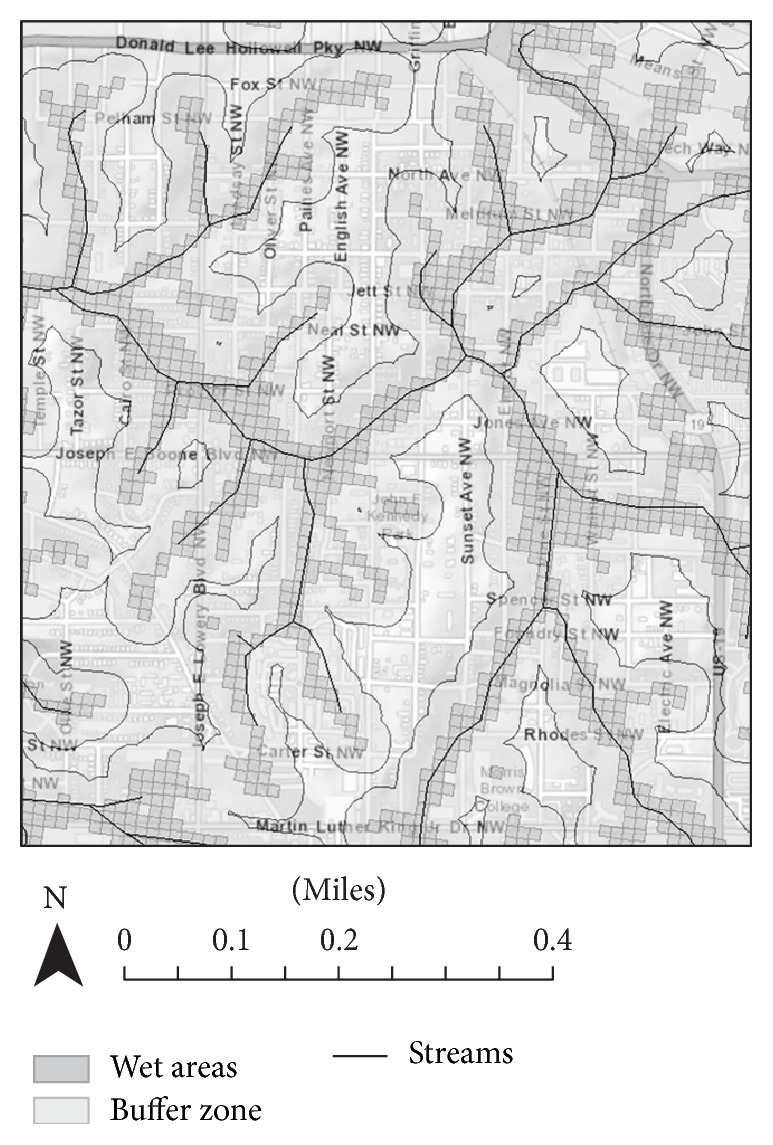
Map of Study Boundaries and Wetness Index [street map is from ArcGIS World Street Map (sources: Esri, HERE, DeLorme, USGS, Intermap, iPC, NRCAN, Esri Japan, METI, Esri China (Hong Kong), Esri (Thailand), MapmyIndia, TomTom©, OpenStreetMap contributors, and the GIS User Community)]. Wetness Index map and stream map were obtained from US EPA.

The two Atlanta communities of English Avenue and Vine City, in the Proctor Creek watershed, are shown in Figure 1. The boundaries of the study communities were Donald Lee Hollowell Parkway to the north, Martin Luther King Drive to the south, Northside Drive to the east, and Temple Street to the west. After dividing this watershed into 30 × 30 m cells using Arc Geographic Information System (ArcGIS™), a topographical analysis identified low-lying cells in the watershed which would be most prone to flooding. A cell was considered “wet,” if it had an EPA Wetness Index [6] value ≥ 500 (values are unscaled). Once wet cells were identified, buffer zones of 70 m around the wet cells were delineated to represent nearby areas not as prone to flooding (Figure 1).

Researchers walked through the communities and documented the address of each home in the wet areas and buffer zone. Global Positioning System (GPS) coordinates for each home were obtained by using a cell phone application to mark the location of each residence in either a wet or buffer cell. If a residence was obviously vacant, it was excluded from consideration (boarded up windows, broken or missing windows, and uncontrolled growth of vegetation were the main indicators used to assess whether a home was abandoned). Among the 1,954 recorded residences, a sample of 507 homes was randomly selected, independent of location in a wet cell or buffer zone.

Each of the two data-collection teams included a public-health graduate student and a resident from each community. These team members were selected through an interview process conducted by Dr. Winquist and Dr. Noibi from a group of four students and four residents (seven students applied). The data-collection teams completed human subjects training. They also went through a mold and indoor environmental-issues awareness training.

Data collection occurred between June and August 2014. The two teams received a list of the selected residences they were to visit. Each residence was visited up to three times, at different times of day, to try to contact an adult resident (aged ≥ 18 years). The survey teams briefly explained the study to the first adult resident contacted. Residents were informed they would receive a $25 gift card, if they agreed to a home inspection and answered the majority of questions in the survey. If the resident expressed interest in participating in the study, informed consent was obtained. When necessary, appointments were made to return at a time more convenient for the resident. The survey teams recorded information about refusals, inability to contact an adult after three attempts, and vacant properties.

The survey included administration of a questionnaire, a home inspection, and the collection of an indoor dust sample. The questionnaire portion of the survey was conducted first and took approximately twenty minutes. The study staff read the questions to the participant and recorded the participant's answers. If requested, a paper version of the survey was given to the participant; however, the survey administrator read each question and manually recorded all responses. After the study questionnaire was completed, the study team and participant walked through the residence to conduct the inspection and collect the dust sample. The home inspection and dust collection took 15 to 30 minutes depending on the size of the residence and the extent of mold or water damage present.

### 2.1. Study Components

On the questionnaire, information was collected about the number of people living in the residence and their ages; the smoking status of the participant and other people living in the residence (including whether or not they smoked inside the residence); whether cockroaches, mice, or rats were present in the residence and how frequently they were observed; the number of years the participant had occupied the residence; whether he or she owned or rented the property; whether there was air conditioning in the residence; whether or not the participant used air conditioning on most days during the summer, if it was present; the history of flooding in the residence (defined as water from outside covering at least a quarter of the floor of a room); and the history of leaks in the residence (defined as plumbing leaks, leaks around tubs or sinks, or leaks that let rain water in) (Table 1). Additional housing-related questions of interest to the community were also included and are not reported here. Health information was also collected for the respondent. The participant was asked to self-report whether a doctor or nurse had ever diagnosed them with certain relevant medical conditions (asthma, chronic obstructive pulmonary disorder, allergies, etc.), whether they currently had those diagnosed conditions, and the frequency of relevant symptoms (wheezing, coughing, running nose, etc.) during the previous month. Analyses relating to asthma focused on self-reports of a diagnosis of asthma (from a doctor or nurse) with a self-report that the asthma was current (referred to as “self-reported current asthma”). Health information was only collected for the individual who completed the survey and not for any other household members.

The home inspection, at a minimum, included the living room, kitchen, a bathroom, and, if possible, the participant's bedroom and, if the home had a basement, it was also inspected. Visual evidences for mold, mice, cockroaches, and pets were noted. The visual inspection for mold and/or water damage did not involve invasive activity, for example, moving furniture. Mold in the bathroom around caulk or on the shower curtain was not included in the analysis, as this was believed to be related to normal usage of the bathroom and cleaning patterns. Mold observed in other areas of the bathroom, such as the ceiling or walls, was recorded and included in the analyses. The location (room and position) and size of any mold growth or water damage were recorded and the area was photographed (if permitted by the participant).

### 2.2. Dust Sample Collection and Testing

For each residence, a single dust sample was collected by wiping the tops of doorways, bookshelves, and other surfaces (other than the floor) using a Swiffer™ Sweeper cloth until the cloth was very gray [7]. The survey team member collecting the dust sample wore a disposable glove to avoid contaminating the sample. Dust samples were typically collected in the living room, kitchen, or bedroom of the study participant. After collection of the dust sample, the cloth was placed in a zippered plastic bag and labeled with the study number. Samples were kept at room temperature throughout collection, transport, and shipping.

### 2.3. Mold Analysis and ERMI Calculation

Each dust sample was sieved (300 *μ*m pore size) and five mg of each sieved dust sample was extracted to recover the DNA, which was then purified using the DNA-EZ kit (GeneRite, Monmouth Junction, NJ). Each of the 36 ERMI molds was quantified by mold specific quantitative PCR (MSQPCR) assays [8]. The standard MSQPCR assay contained 12.5 *μ*L of “Universal Master Mix” (Applied Biosystems Inc., Foster City, CA), 1 *μ*L of a mixture of forward and reverse primers at 25 *μ*M each, 2.5 *μ*L of a 400 nM TaqMan probe (Applied Biosystems Inc.), 2.5 *μ*L of 2 mg/mL fraction V bovine serum albumin (Sigma Chemical, St. Louis, MO), and 2.5 *μ*L of DNA free water (Cepheid, Sunnyvale, CA). To this mix was added 5 *μ*L of the DNA extract from the sample. The ERMI value in each home was then calculated, as described below.

The ERMI metric classifies the 36 indicator mold species into two groups. Group 1 includes 26 species and indicates water damage. Group 2 includes ten species which are commonly found in homes across the United States, even without water damage, and come primarily from outdoors (shown in the following list) [9].

*Environmental Relative Moldiness Index (ERMI) Indicator Molds*. Group 1 associated with water-damaged homes and Group 2 is common in homes, independent of water damage.

*Group 1*

*Aspergillus flavus *
*Aspergillus fumigatus *
*Aspergillus niger *
*Aspergillus ochraceus*
*Aspergillus penicillioides*
*Aspergillus restrictus *
*Aspergillus sclerotiorum *
*Aspergillus sydowii *
*Aspergillus unguis *
*Aspergillus versicolor *
*Aureobasidium pullulans *
*Chaetomium globosum *
*Cladosporium sphaerospermum *
*Eurotium amstelodami *
*Paecilomyces variotii *
*Penicillium brevicompactum *
*Penicillium corylophilum *
*Penicillium crustosum *group
*Penicillium purpurogenum *
*Penicillium spinulosum *
*Penicillium variabile *
*Scopulariopsis brevicaulis*
*Scopulariopsis chartarum*
*Stachybotrys chartarum *
*Trichoderma viride*
*Wallemia sebi. *

*Group 2*

*Acremonium strictum*
*Alternaria alternata*
*Aspergillus ustus*
*Cladosporium cladosporioides *1
*Cladosporium cladosporioides *2
*Cladosporium herbarum*
*Epicoccum nigrum*
*Mucor *group
*Penicillium chrysogenum*
*Rhizopus stolonifer.*

The ERMI calculation takes the results from the concentrations (cells/mg dust) of each of 36 molds and mathematically converts these into a single number as follows:

(1) $$\begin{array}{c} ERMI=\overset{26}{\underset{i=1}{\sum }}log_{10}\left(\;s_{1i}\;\right)-\overset{10}{\underset{j=1}{\sum }}log_{10}\left(\;s_{2j}\;\right). \end{array}$$

The concentration of each of the 26 Group 1 molds (*s*_1*i*_) is converted to a log and then the “Sum of the Logs of Group 1” (SLG1) molds is determined. Similarly, the concentration of each of the ten Group 2 molds (*s*_2*j*_) is converted to a log and then the “Sum of the Logs of Group 2” (SLG2) molds is determined. The arithmetic difference (SLG1-SLG2) is the ERMI value for the home [9].

The ERMI scale runs from about −10 to about 30 and is divided into quartiles. For example, 25% of homes in the US have an ERMI value below −4 and are in the lowest relative mold contamination quartile and the 25% of homes with ERMI values above 5 are in the highest relative mold contamination quartile [9].

### 2.4. Statistical Analyses

Bivariate analyses were conducted to assess the association between (1) observed mold and ERMI values; (2) observed mold and self-reported current asthma; and (3) ERMI values and self-reported current asthma. A two-sided *t*-test was used to assess the statistical significance of the crude associations between observed mold and ERMI values and between self-reported current asthma and ERMI values. A chi-square test was used to assess the statistical significance of the crude association between observed mold and self-reported current asthma. Associations were considered statistically significant if *P* ≤ 0.05.

After bivariate analyses, two separate logistic regression models were fit to further explore the relationship between self-reported current asthma and each target exposure, that is, ERMI values (included as a linear term in the model) or observed mold. Variables considered as potential confounders in the logistic regression models included indoor smoking, air conditioning use, pets, cockroaches, mice, the duration of time the participant had lived in the residence, and the number of people living at the residence. Variables were removed from the final model if their removal did not change the effect estimate for the exposure of interest by more than 10%. Because we hypothesized that the observed association between our measures of mold and self-reported current asthma could differ depending on how long a person had lived at their residence, we also considered models that were stratified based on whether the respondent had lived at their current residence for two years or less (the two-year cut point was chosen to be close to the median value of 2.5 years and consistent with the typical annual renewal of a lease).

## 3. Results

**Figure 2:**
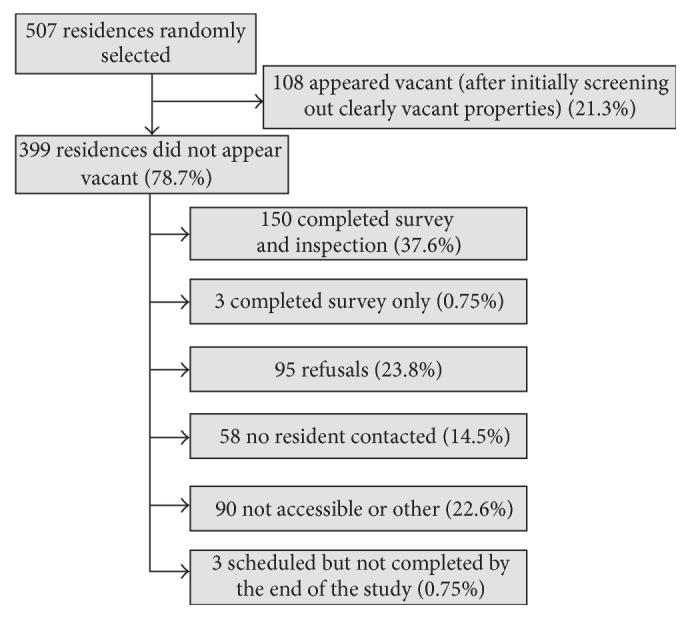
Diagram of recruitment process with frequencies of survey response and reasons for nonresponse.

Of the 507 selected residences, 399 (79%) did not appear vacant at the time of survey administration. Among the 399 nonvacant residences, 153 (39%) residents completed the questionnaire and 150 also allowed the home inspection and dust sample collection, as shown in Figure 2. Table 1 summarizes the characteristics of the study population and homes. The median number of people living at each residence was two and the median number of years respondents had lived at their current residence was 2.5 years. The majority of respondents (76%) were renters and the majority lived in a house (53%). Of those living in houses, 30% reported having a basement.

Of the residences with participants, 34% were located in wet areas (based on the Wetness Index) and the rest were in the buffer zone. Twelve percent of respondents reported a history of flooding in their current residence while they had lived there and only 3% reported knowing of flooding in the residence before they had lived there. However, 46% of respondents reported experiencing at least one water leak (roof, pipe, window, etc.).

Mold was observed by the survey team in 35% of the homes. In bivariate analyses, homes with observed mold were more likely to have basements (OR = 3.17, 95% CI: 1.26–8.04) and resident-reported water leaks (OR = 3.03, 95% CI: 1.51–6.08) than homes without observed mold. The odds of a study participant self-reporting having current asthma was not significantly different in homes with or without observed mold.

**Figure 3:**
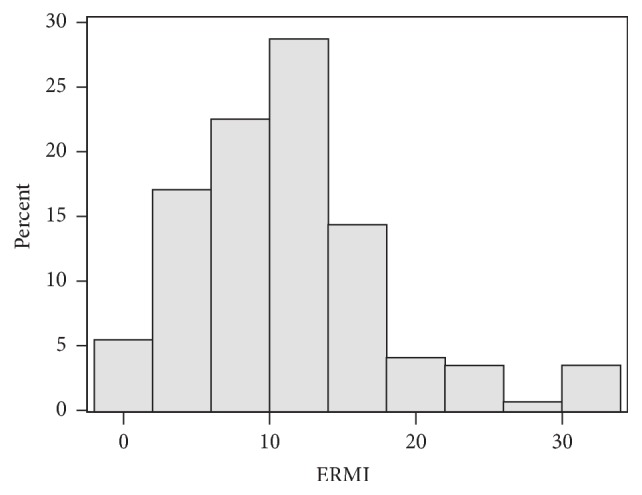
Distribution of Environmental Relative Moldiness Index (ERMI) values for the homes (*n* = 150) in the English Avenue and Vine City communities in Atlanta, Georgia.

The median ERMI value for all homes (Figure 3) was 10.9 and 83% of the homes had ERMI values above 5. The mean ERMI value for homes with observed mold (13.97) was significantly greater (two-sided *t*-test, *P* < 0.05) than the mean ERMI value for homes without observed mold (9.55). In bivariate analysis, participants who self-reported having current asthma (*n* = 21) had higher mean ERMI values (13.5) than participants (*n* = 125) not self-reporting current asthma (10.7). This difference was not statistically significant (two-sided *t*-test, *P* = 0.08).

The results of the logistic regression models are shown in Tables 2 and 3. The final models controlled for indoor smoking and duration of residence. For the logistic regression model for self-reported current asthma in relation to observed mold (controlling for indoor smoking and duration of residence), there was no significant relationship between observed mold and current asthma (Table 2). For the logistic regression model for self-reported current asthma in relation to ERMI values (controlling for indoor smoking and duration of residence), ERMI values were positively, but not significantly, associated with self-reported current asthma (OR = 1.05, 95% CI: 0.98–1.13) (Table 3). However, in logistic regression models (controlling for indoor smoking), the homes' ERMI values were positively and significantly associated with self-reported current asthma (adjusted OR per unit increase in ERMI = 1.12, 95% CI: 1.01–1.25; two-tailed *P* = 0.04) (Table 3) for participants residing at their current residence for two years or less.

## 4. Discussion

Although the impact of flooding was the initial concern in the communities, the prevalence of current residents' reports of flooding was much lower (only 12%) than expected. The English Avenue and Vine City communities have historically experienced frequent flooding due to rain-water runoff and sewer overflows and because impervious surfaces cover 33% of the Proctor Creek watershed [10]. However, many of the homes closest to the creek had already been abandoned by the time of this study. At this time, water leaks, condensation, poor ventilation, and poor humidity control seem to be the major sources of home water damage. The high proportion of residents reporting water leaks and the resulting increased chances for mold growth might be due to the fact that most of the study participants were renters with little control over timely home maintenance.

The prevalence of self-reported current adult-asthma in the English Avenue and Vine City communities of Atlanta, GA, was 14% compared to 8.4% for the state of Georgia and 7.4% for the US adult population [11]. Visible mold was observed in 35% of these homes compared to only 15.2% of homes in a survey of low-income housing in Boston [12]. In addition, 83% of the study homes had ERMI values above 5 compared to only 25% of US homes nationally [9]. The high levels of mold contamination, as described by the high ERMI values, may be contributing to the high prevalence of self-reported current asthma. Homes with high ERMI values, like those found in these communities, have been associated with asthma development and current asthma in a review of six epidemiological studies of asthma in water-damaged, moldy homes [13].

The association between ERMI values and self-reported current asthma in this study was found only for those living in their homes for two years or less. Because this was a cross-sectional study and the date of the asthma diagnosis was not collected, the temporal relationship between the diagnosis of asthma and residence in the participants' current homes is not known. It is possible that people who have asthma may move into certain homes and soon realize that the conditions are aggravating their asthmatic symptoms, leading them to move out within a few years. Therefore, participants who lived in a residence with mold for more than two years may be a self-selected population that is less sensitive to mold.

An important limitation of the study was our inability to measure a comprehensive set of exposures that might be important causes of the reported asthma. It is possible that the associations we observed between mold or ERMI values and self-reported current asthma could be confounded by such unmeasured factors, particularly unmeasured factors associated with damp environments. We were able to consider several factors as potential confounders, including indoor smoking, air conditioning use, the duration of time a person had lived at the residence, the number of people living at the residence, and the presence of mice, cockroaches, and pets. Our models controlled for smoking indoors and duration of residence, as these were the only exposures for which inclusion in the model affected the estimates for the exposures of interest. However, other possibly relevant exposures, for example, endotoxins, dust mites, pollen, were not measured and the observed mold and high ERMI values may only be coincidental markers for other exposures that are the real cause(s) of the residents' asthma. The study was also limited by lack of assessment of asthma severity. A strength of this study was that local residents participated as active members of the investigative team. Occupant access and cooperation were improved by having a team of residents and researchers.

Although this study does not establish the fact that the mold exposures in these communities caused the high prevalence of asthma, many previous studies have found mold exposure and damp buildings to be associated with asthma morbidity [14–18]. Therefore, the high prevalence of mold contamination in the homes in these communities should be a public health concern. In a recent review, the national economic cost of indoor dampness and mold was estimated at $16.8 billion per year for asthma morbidity and mortality [19]. Landlords, public health and code enforcement agencies, city, county, or state legislatures or councils, and the municipal sewer district may all have a role in efforts to control conditions that can lead to mold such as flooding and poor housing conditions.

## 5. Conclusions

Documentation of the environmental exposures and the prevalence of asthma in these communities has been presented to the residents and local officials during a series of three public meetings. Community-based organizations have taken responsibility for planning and implementing activities in response to the study findings. This kind of community-partnered research could be a model for other communities.

## Figures and Tables

**Table 1:** Characteristics of survey respondents and their residences.

		Number	Percentage
Demographics	Gender (*N* = 153)		
	Female	89	58
	Male	64	42
	Renter/owner (*N* = 149)		
	Rent	113	76
	Own	36	24
	Age (*N* = 152)	Median: 47	Range: 21–88

Residence characteristics	Location of residence (*N* = 153)		
	Wet area	52	34
	Buffer zone	101	66
	Type of residence (*N* = 153)		
	Individual house	81	53
	Basement	24	30
	No basement	55	68
	Unknown basement status	2	2
	Apartment/condo	54	35
	Duplex	5	3
	Townhouse	13	8
	Duration at residence (years) (*N* = 153)	Median: 2.5	Range: <1–84
	Number of people in residence (*N* = 153)	Median: 2	Range: 1–12

Environmental characteristics in residence	Observed mold (*N* = 150)		
	Yes	53	35
	No	97	65
	Leaks (*N* = 153)		
	Yes	71	46
	No	82	54
	Flooding (*N* = 153)		
	Yes, while the participant lived in the residence	19	12
	Yes, before the participant lived in the residence	4	3
	No	130	85
	Mice (*N* = 153)		
	Reported mice	15	10
	No reported mice	138	90
	Cockroaches (*N* = 153)		
	Yes	51	33
	No	102	67
	Pets (*N* = 153)		
	Yes	49	32
	No	104	68
	Smoking inside residence (*N* = 153)		
	Yes	59	39
	No	94	61
	Air conditioning use on most days during the summer (*N* = 152)		
	Yes	98	64
	No	54	36
	ERMI values (*N* = 146)	Mean: 11.12 Median: 10.9	Range: −1.85–32.02

Reported health characteristics	Self-reported current asthma (*N* = 152)		
	Yes	22	14
	No	130	86

**Table 2:** Results of logistic regression models examining the association between observed mold and self-reported current asthma. Odds ratios are presented for all variables included in the final models, which included observed mold, smoking inside the residence, and residence duration (in the unstratified model).

	Unstratified model				Duration of residence				Duration of residence			
					≤2 years; 73 observations				>2 years; 76 observations			
	Odds ratio	95% confidence intervals		*P* value	Odds ratio	95% confidence intervals		*P* value	Odds ratio	95% confidence intervals		*P* value
Observed mold	0.82	0.30	2.26	0.69	1.18	0.30	4.68	0.81	0.59	0.13	2.64	0.49
Any smoking inside residence	1.81	0.71	4.60	0.21	3.80	1.04	13.98	0.04	0.61	0.11	3.25	0.56
Residence duration (per 1-year increase)	1.46	0.56	3.80	0.44	—	—	—	—	—	—	—	—

**Table 3:** Results of logistic regression models examining the association between ERMI values and self-reported current asthma. Odds ratios are presented for all variables included in the final models, which included the ERMI value (included as a linear term in the model), smoking inside the residence, and residence duration (in the unstratified model).

	Unstratified model				Duration of residence				Duration of residence			
					≤2 years; 70 observations				>2 years; 76 observations			
	Odds ratio	95% confidence intervals		*P* value	Odds ratio	95% confidence intervals		*P* value	Odds ratio	95% confidence intervals		*P* value
ERMI	1.05	0.98	1.13	0.14	1.12	1.01	1.25	0.04	1.01	0.92	1.12	0.79
Any smoking inside residence	1.67	0.64	4.32	0.29	5.11	1.16	22.57	0.03	0.49	0.09	2.64	0.40
Residence duration (per 1-year increase)	1.46	0.57	3.77	0.43	—	—	—	—	—	—	—	—

## References

[1] Moorman J. E., Rudd R. A., Johnson C. A. (2007). National surveillance for asthma—United States, 1980–2004. *Morbidity and Mortality Weekly Report. Surveillance Summaries*.

[2] Centers for Disease Control and Prevention *Trends in Asthma Prevalence, Health Care Use, and Mortality in the United States, 2001–2010*.

[3] Persky V., Turyk M., Piorkowski J. (2007). Chicago Community Asthma Prevention Program. Inner-city asthma: the role of the community. *Chest*.

[4] Centers for Disease Control and Prevention (2013). CDC health disparities and inequalities report—United States. *Morbidity and Mortality Weekly Report*.

[5] Beasley R., Semprini A., Mitchell E. A. (2015). Risk factors for asthma: is prevention possible?. *The Lancet*.

[6] Sørensen R., Zinko U., Seibert J. (2006). On the calculation of the topographic wetness index: evaluation of different methods based on field observations. *Hydrology and Earth System Sciences*.

[7] Vesper S., Prill R., Wymer L., Adkins L., Williams R., Fulk F. (2015). Mold contamination in schools with either high or low prevelance of asthma. *Pediatric Allergy and Immunology*.

[8] Haugland R. A., Vesper S. J. Identification and quantification of specific fungi and bacteria.

[9] Vesper S. J., McKinstry C., Haugland R. A. (2007). Development of an environmental relative moldiness index for homes in the U.S. *Journal of Occupational and Environmental Medicine*.

[10] Adkins L., Baugh T., Egetter D., Fulk F. (2015). Proctor Creek's boone boulevard green street project Health Impact Assessment (HIA), Atlanta, Georgia.

[11] Centers for Disease Control and Prevention Asthma Surveillance Data. http://www.cdc.gov/asthma/asthmadata.htm.

[12] Adamkiewicz G., Spengler J. D., Harley A. E. (2014). Environmental conditions in low-income urban housing: clustering and associations with self-reported health. *American Journal of Public Health*.

[13] Vesper S., Wymer L. (2016). The relationship between environmental relative moldiness index values and asthma. *International Journal of Hygiene and Environmental Health*.

[14] World Health Organization Europe (2009). *WHO Guidelines for Indoor Air Quality: Dampness and Mould*.

[15] Quansah R., Jaakkola M. S., Hugg T. T., Heikkinen S. A., Jaakkola J. J., Behrens T. (2012). Residential dampness and molds and the risk of developing asthma: a systematic review and meta-analysis. *PLoS ONE*.

[16] Mendell M. J., Mirer A. G., Cheung K., Tong M., Douwes J. (2011). Respiratory and allergic health effects of dampness, mold, and dampness-related agents: a review of the epidemiologic evidence. *Environmental Health Perspectives*.

[17] Mazur L. J., Kim J. (2006). Committee on Environmental Health. Spectrum of noninfectious health effects from molds. *Pediatrics*.

[18] Norbäck D., Zock J.-P., Plana E. (2013). Mould and dampness in dwelling places, and onset of asthma: the population-based cohort ECRHS. *Occupational and Environmental Medicine*.

[19] Mudarri D. H. (2016). Valuing the economic costs of allergic rhinitis, acute bronchitis, and asthma from exposure to indoor dampness and mold in the US. *Journal of Environmental and Public Health*.

